# Influence of the Onset of Menopause on the Risk of Developing Alzheimer’s Disease

**DOI:** 10.7759/cureus.69124

**Published:** 2024-09-10

**Authors:** Gabriela Briceno Silva, Joanne Arvelaez Pascucci, Hajira Karim, Gurpreet Kaur, Ricardo Olivas Lerma, Apindervir Kaur Mann, Sulochana Gnanasekaran, Karem D Thomas Garcia

**Affiliations:** 1 Obstetrics and Gynecology, Universidad de Oriente, Barcelona, VEN; 2 Infectious Disease, Universidad Central de Venezuela, Caracas, VEN; 3 Internal Medicine, Istanbul Medipol University, Istanbul, TUR; 4 Neurosurgery, Institute of Human Behaviour and Allied Sciences, New Delhi, IND; 5 General Practice, Universidad Autonoma de Chihuahua, Chihuahua, MEX; 6 General Practice, Government Medical College Amritsar, Amritsar, IND; 7 Internal Medicine, New York Medical College, St. Mary's and St. Clare's Hospital, Passaic, USA; 8 General Practice, Universidad de Oriente, Barcelona, VEN

**Keywords:** hormone replacement therapy, menopause management, cognitive decline, alzheimer's disease, menopause

## Abstract

Menopause is a natural phase marked by the permanent cessation of menstrual cycles, occurring when the production of reproductive hormones from the ovaries stops for at least 12 consecutive months. Studies have suggested a potential connection between menopause and a heightened risk of developing Alzheimer's disease (AD), underscoring the significant role of reduced estrogen levels in the development of AD. Estrogen plays a crucial role in brain metabolism, influencing energy metabolism, synaptic plasticity, and cognitive functions. The cognitive benefits associated with hormone replacement therapy (HRT) are believed to be linked to estrogen's neuroprotective effects, either through direct action on the brain or indirectly by improving cardiovascular health. Extensive literature supports the positive impact of estrogen on brain cells. While the physiological effects of estrogen on the brain have not been consistently replicated in clinical trials, further research is crucial to provide more definitive recommendations to menopausal patients regarding the influence of HRT on AD. This review aims to comprehensively explore the interplay between menopause and AD, as well as the potential of HRT to mitigate cognitive decline in post-menopausal individuals.

## Introduction and background

Menopause is a normal condition involving the permanent end of menstrual cycles due to the cessation of the production of reproductive hormones from the ovaries for at least 12 consecutive months [1]. Perimenopause is an ill-defined period that surrounds the final years of a woman’s reproductive life. It begins with the first onset of menstrual irregularity and ends after one year of amenorrhea has occurred. There are two stages to this menopausal transition: the early transition, where cycles are mostly regular, with relatively few interruptions, and the late transition, where amenorrhea becomes more prolonged and lasts for at least 60 days, up to the final menstrual period [2,3].

Most women experience vasomotor symptoms, but menopause can affect many different organ systems [1]. The decline of estradiol (E2) typical of menopause has been associated with changes in the brain, including cognitive changes, sleep effects, and mood effects [4]. E2 interacts with the cholinergic, dopaminergic, and mitochondrial functions, has effects on brain volumes and neuronal connectivity, and has shown interactions in neuropsychiatric disorders, including Alzheimer’s disease (AD), schizophrenia, and depression [5].

AD is a progressive neurodegenerative disorder that accounts for more than half of all dementia cases in the elderly [6]. It remains the fifth-leading cause of death among Americans aged 65 years and older [7]. Women comprise a disproportional two-thirds of all AD cases. Evidence suggests a link between menopause and a higher risk of developing AD, highlighting the critical role of decreased estrogen levels in AD pathogenesis [6]. Elucidating this connection is crucial for better understanding and developing targeted therapeutic interventions, such as hormone replacement therapy (HRT), to mitigate AD risk and for devising lifestyle, dietary, and other guidelines that help maintain cognitive health throughout the menopausal transition and beyond [8].

The present review is organized to explore the relationship between menopause and AD comprehensively. Initially, we examine the stages of menopause and their associated hormonal changes. Then, we review the pathophysiological mechanisms that might link menopause to AD. Further sections delve into brain imaging modalities and biomarkers pertinent to AD in the menopausal context, the impact of HRT on cognitive function and AD prevention, and lifestyle interventions that may diminish AD risk. Concluding sections will illuminate future research directions and underscore existing gaps in the current knowledge base.

## Review

Menopause stages and hormonal changes

The stages of menopause (perimenopause, menopause, and post-menopause) entail distinct physiological and hormonal changes that substantially affect a woman's health and well-being. Perimenopause, also referred to as the menopausal transition, is the phase leading up to menopause when a woman's body begins to undergo hormonal shifts. This period can last from a few months to several years, typically commencing in a woman's 40s but sometimes starting as early as the 30s in cases of premature ovarian insufficiency [9]. Fluctuations in estrogen and progesterone levels occur irregularly due to diminished ovarian function and progressive follicle depletion, resulting in menstrual cycle irregularities. Women may encounter various symptoms, including hot flashes, night sweats, and changes in sexual function [10]. Some may also notice changes in skin elasticity and bone density (see Table 1 for closer detail) [11]. Collectively, these symptoms, which impact social and psychological well-being, are believed to contribute to developing mood disorders and sleep disturbances [12].

**Table 1:** Key characteristics of the stages of menopause.

	Perimenopause	Menopause	Postmenopause
Hormonal changes, follicle-stimulating hormone (FSH), and ovarian function [11]	Fluctuating estrogen and progesterone levels; FSH levels rise due to decreased ovarian function; ovarian function declines, leading to irregular ovulation and hormone production	Significant drop in estrogen; FSH levels peak due to deficient estrogen; ovarian function ceases, no ovulation, minimal hormone production	Low, stable estrogen levels; FSH levels remain high due to consistently low estrogen; ovaries no longer produce significant amounts of hormones
Symptoms [12]	Irregular periods, hot flashes, night sweats, sleep disturbances, mood swings, decrease in memory and focus	Hot flashes, night sweats, urogenital symptoms such as vaginal dryness and sexual problems, mood swings, and depression	Reduced frequency of hot flashes, continued vaginal dryness, and urogenital symptoms.
Age range [11]	Late 30s to early 50s	The average onset age is around 51 years	The 50s and beyond
Duration [11]	Typically, 4-8 years	Defined as 12 consecutive months without a menstrual period	It begins 12 months after the last menstrual period and continues indefinitely
Health risks [11]	Increased risk of osteoporosis and cardiovascular disease begins	Heightened osteoporosis and cardiovascular risk due to lower estrogen levels	Continued increased risk of osteoporosis and cardiovascular disease

The exact cause of the increase in core body temperature during the late menopausal transition is not fully understood, and estrogen changes alone do not fully explain vasomotor symptoms. It is believed that the disruption in temperature regulation is centrally mediated, involving alterations in the hypothalamic beta-endorphin activity and changes in adrenergic, muscarinic, and nicotinic receptors, as well as serotonin and noradrenaline, which are thought to affect temperature control within the hypothalamus [13].

Menopause is defined as the point in time when a woman has not had a menstrual period for 12 consecutive months. The average age of menopause is around 51 years in the United States, but it can occur naturally anywhere between ages 45 and 55 [1]. Estrogen and progesterone levels drop significantly, leading to the cessation of ovulation and menstruation [14]. Common symptoms during menopause include hot flashes, night sweats, vaginal dryness, mood changes, and decreased libido. Estrogens stimulate vaginal epithelial cells to produce glycogen, which lactobacilli convert into lactic acid, keeping the vaginal pH around 3.5-4.7. During hypoestrogenism, glycogen production drops, reducing lactobacilli and raising vaginal pH. This condition leads to thinner vaginal epithelium and increased pH. Additionally, hypoestrogenism causes a reduction in epithelial cell layers and degeneration of collagen and elastin fibers in the connective tissue, resulting in decreased tissue elasticity and increased mucosal fragility [15].

The term postmenopause refers to the years following menopause, starting from the point after a woman has gone 12 months without a menstrual period. Estrogen levels remain low, and the body adjusts to this new hormonal baseline. While some menopausal symptoms may subside, others, like vaginal dryness and osteoporosis risk, can persist or worsen. Women may also experience urinary incontinence, weight gain, and changes in skin texture. Monitoring bone density and cardiovascular health is crucial, as osteoporosis and heart disease risk increase during postmenopause. The Framingham study found that natural menopause before age 42 is linked to a 103% increased risk of ischemic stroke compared to later menopause, with the risk rising as menopause occurs earlier. Among 2,873 women followed for 24 years, none of the premenopausal women had myocardial infarctions or died from cardiovascular disease (CVD). Still, postmenopausal women experienced over twice as many cardiovascular events compared to premenopausal women (70 vs. 20 events). While further research is needed to clarify the precise mechanisms behind endothelial dysfunction and significant artery stiffening, it is believed that oxidative stress, vascular inflammation, estrogen receptor alpha issues, and endothelial nitric oxide synthase (NOS) dysfunction all play a role in these processes [16-18].

Previous studies have shown that higher estrogen levels are positively associated with bone mineral density (BMD) and help prevent osteoporotic fractures. This relationship is likely due to estrogen's direct effects on osteoblasts, osteocytes, and osteoclasts, which help balance bone formation and resorption [19].

Neurological Symptoms During Menopause

During menopause, women frequently experience various neurological symptoms, including hot flashes, which lead to intense heat and sweating; sleep disturbances, such as difficulty falling and staying asleep; and cognitive decline, affecting memory and concentration. Mood swings, headaches or migraines, and dizziness or vertigo are common, significantly impacting daily life and emotional well-being [20,21]. These symptoms are closely associated with hormonal fluctuations, particularly decreasing estrogen levels. Factors like age, socioeconomic status, and pre-existing health conditions can exacerbate these issues, highlighting the need for personalized treatment plans. Often, managing and alleviating these symptoms effectively requires lifestyle changes, including adjustments to diet, exercise, and targeted medical interventions [22].

Studies indicate that women in the late stage of menopausal transition or early postmenopause with elevated anxiety levels tend to experience the most severe hot flashes. Individual analyses have revealed that more severe hot flashes are significantly associated with lower estrone levels, higher follicle-stimulating hormone (FSH) levels, higher body mass index (BMI), higher cortisol levels, and younger age at the onset of the late menopausal transition stage [23]. Prolonged high cortisol levels, as observed in women with severe hot flashes, are linked to cognitive decline [24]. Evidence suggests that estrogen and its alpha isoform receptor act as neuro-anti-inflammatories. When estrogen levels decrease as they do during menopause, neuro-inflammatory processes persist. Decreased estrogen in microglia may influence the onset and progression of neurodegenerative diseases [25].

It is crucial to note that the menopausal transition is connected to a higher risk of dementia due to post-menopausal estrogen loss and modifiable factors like hypertension, diabetes, depression, and physical inactivity [26]. These interactions seem to contribute to a higher incidence of dementia in postmenopausal women compared to men.

Pathophysiological mechanisms linking menopause and Alzheimer’s disease

**Figure 1:**
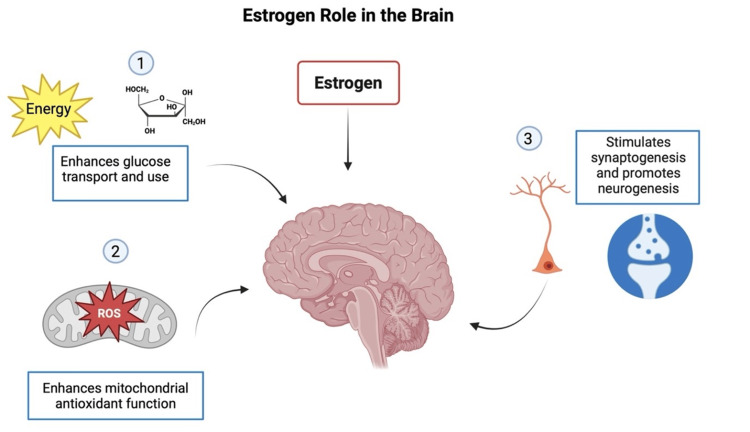
Estrogen's role in the brain. 1. Estrogen enhances glucose transport and utilization in the brain. 2. Estrogen improves mitochondrial antioxidant function. 3. Estrogen has neuroprotective effects on the brain by stimulating neurogenesis and synaptogenesis [27]. ROS: reactive oxygen species. Figure recreated with information from the cited source.

Reductions in estrogen levels during menopause significantly impact brain function, leading to amyloid-beta accumulation, neuroinflammation, and reduced metabolic activity, among other effects. Estrogen is crucial in maintaining cognitive health by influencing glucose metabolism, neuronal plasticity, oxidative stress, and reducing inflammation [4]. All these mechanisms are explained in the following sections and summarized in Figure 1, recreated with relevant information from cited sources.

Implications of Estrogen in Brain Function

Estrogen plays a crucial role in brain function, influencing various processes such as energy metabolism, synaptic plasticity, and cognitive functions. It enhances glucose transport and utilization, essential for maintaining brain energy levels [28]. This hormone acts through estrogen receptors, abundantly present in regions like the hippocampus and prefrontal cortex, which are critical for memory and cognitive processes [29]. Estrogen stimulates synaptogenesis, the growth of synapses between neurons, and promotes synaptic transmission, thereby supporting learning and memory. Additionally, estrogen regulates mitochondrial function, improving efficiency and enhancing the brain's antioxidant defense [27]. This reduces oxidative stress, a critical factor in neurodegeneration, and promotes neuronal survival and function. The neuroprotective effects of estrogen also include the reduction of neuroinflammation, the promotion of neurogenesis, and the birth of new neurons, further underscoring its importance in brain health [30]. Research suggests that the fluctuation and decline of estrogen levels during menopause may predispose women to AD. Low estrogen levels are linked to reduced glucose metabolism, increased amyloid-beta deposition, and heightened neuroinflammation, all of which contribute to AD pathology. Although a direct causal link has not been definitively established, the evidence highlights the crucial role of estrogen in maintaining brain health and its potential impact on the development of AD [4].

Amyloid-Beta Deposition

The connection between menopause, decreased estrogen, and increased amyloid-beta (Aβ) deposition is a well-documented pathway in AD pathology [31]. Estrogen influences the processing of amyloid precursor protein (APP), reducing the production of Aβ peptides by favoring non-amyloidogenic pathways. Without sufficient estrogen, the balance shifts toward amyloidogenic processing, increasing the production and accumulation of Aβ peptides [32]. These peptides aggregate to form amyloid plaques, a hallmark of AD. Moreover, estrogen enhances the clearance of Aβ through the activation of microglia and upregulation of enzymes that degrade Aβ peptides. With the decline in estrogen during menopause, these protective mechanisms are diminished, leading to the accumulation of neurotoxic Aβ plaques. This accumulation disrupts synaptic function, triggers inflammatory responses, and contributes to neuronal death, thereby driving the progression of AD [33].

Neuroinflammation

Neuroinflammation plays a central role in the development of AD, and the decline in estrogen levels during menopause further amplifies this inflammatory response. Estrogen exhibits potent anti-inflammatory properties by regulating the function of microglia and astrocytes, the primary immune cells in the brain. It suppresses the production of pro-inflammatory cytokines such as interleukin 1 beta (IL-1β), tumor necrosis factor-alpha (TNF-α), and interleukin 6 (IL-6) while promoting the release of anti-inflammatory cytokines [34,35]. Reduced estrogen levels during menopause lead to increased activation and proliferation of microglia and astrocytes, resulting in heightened inflammation characterized by the release of pro-inflammatory cytokines, oxidative stress, and neurotoxicity. Chronic neuroinflammation causes damage to neurons and synapses and stimulates the deposition of Aβ and hyperphosphorylation of tau, both critical features of AD. This sustained inflammatory environment accelerates neurodegenerative processes, contributing to cognitive decline and the progression of AD [36].

Brain imaging and biomarkers

Recent neuroimaging studies have provided compelling evidence of a strong connection between menopause and an increased risk of AD-related changes in women. Various imaging techniques, such as MRI, functional MRI (fMRI), amyloid positron emission tomography scan (amyloid PET), and tau-targeted PET (tau PET), consistently show that the menopausal transition is associated with significant changes in brain structure and function that are typical of AD. These brain changes manifest as alterations in biomarkers such as grey and white matter volume, white matter hyperintensities, reduced glucose metabolism, beta-amyloid, and tau plaques [37].

Postmenopausal women account for 70% of the population affected by AD [38]. Beta-amyloid deposition, a key AD characteristic, is increased in peri- and post-menopausal women, even during the preclinical stages [39]. Another hallmark pathology of AD, tau neurofibrillary tangles, is more prevalent in older women compared to age-matched men. In females with high beta-amyloid levels, early menopause and delayed initiation of HRT were associated with increased tau deposition compared to age-matched males [40]. These tau neurofibrillary tangles begin to form early in the disease process in Alzheimer's patients. Still, they are only easily detectable later as a disease progression biomarker [41]. The imaging techniques used to visualize these markers are amyloid PET and tau PET.

During the transition from perimenopause to menopause, the brain's metabolism significantly shifts. As previously mentioned, this shift involves reduced glucose metabolism and adenosine triphosphate (ATP) production, resulting in decreased brain activity. These metabolic changes are considered to be indicators of the heightened risk of Alzheimer's in women [42]. Additionally, in individuals with AD, these changes signify early neuronal dysfunction. Altered metabolism can be identified using fluorodeoxyglucose PET (FGD-PET), a commonly used imaging technique for detecting changes in glucose metabolism [43].

Studies have shown that premenopausal women may experience some neuronal loss compared to men in the same age group [44]. Menopausal women demonstrate a notable decrease in grey matter and white matter volume in specific brain areas, such as the frontal, posterior cingulate, medial temporal, and parietal cortex [45]. An essential indicator of compromised brain health is the presence of white matter hyperintensities (WMH), which form due to reduced blood supply to the brain and are often associated with small vessel occlusion and vasomotor symptoms observed in menopause. Research indicates that women post-menopause tend to have a higher burden of WMH compared to premenopausal women [45].

The most effective imaging techniques for studying volume changes are structural MRI and MRI diffusion tensor imaging (MRI-DTI). MRI-DTI provides a three-dimensional view of water molecule diffusion in brain tissue and can measure fractional anisotropy (FA) to assess white matter tracts. Research has found lower FA values in perimenopausal women than their healthy counterparts, indicating changes in white matter microstructure [46].

On the other hand, while MRI can detect WMH, fluid-attenuated inversion recovery (FLAIR) is particularly useful for identifying periventricular and deep white matter hyperintensities by enhancing the visibility of brain lesions through high-contrast images that suppress cerebrospinal fluid (CSF) signal. These changes have been linked to cognitive decline and disease progression in patients diagnosed with AD [47]. While the changes are observed during menopause, further research is needed to determine whether they are simply signs of neurobiological aging or indicate a predisposition to AD later in life to understand the mechanisms underlying this connection fully and to develop targeted interventions for women during this critical period.

For AD findings, both structural and functional MRI are highly sensitive, with a 70-85% success rate in detecting early changes in the brain, such as atrophy or decreased brain activity. However, they lack specificity (65-70%) because degenerative brain changes can also be observed in other neurodegenerative and psychiatric conditions. The most accurate test, with a specificity and sensitivity rate of 80-95%, is the florbetapir-PET scan, which detects amyloid. This is particularly useful in the preclinical stages for identifying Alzheimer's. Tau PET also boasts an 80-95% sensitivity and specificity, but it is typically employed in the later stages of Alzheimer's [47].

Recent research has also highlighted significant biomarker and brain imaging differences between men and women in AD. Biomarkers such as Aβ deposition and tau pathology exhibit distinct patterns across genders, influencing disease manifestation and progression. Studies indicate that women tend to show higher levels of Aβ plaques than men, particularly in regions crucial for memory and cognition, such as the hippocampus and entorhinal cortex [48]. Additionally, women often present with more widespread tau deposition in the later stages of the disease, impacting functional connectivity and neuronal integrity differently than in men [44]. Neuroimaging findings complement these gender-specific differences in biomarkers. For instance, structural MRI studies reveal more significant gray matter atrophy in specific brain regions in women, potentially contributing to differences in clinical presentation and cognitive decline compared to men [49].

Hormone replacement therapy and cognitive function

HRT is a standard treatment to help women manage the symptoms of menopause. It aims to restore hormonal levels to premenopausal status and is also beneficial for the long-term effects of menopause, which include osteoporosis and CVD [50]. HRT effects are determined by the timing of initiation according to age and time since menopause, underlying health status, and duration of therapy, so the treatment needs to be individualized [51].

There are two main types of HRT: estrogen therapy and combination therapy (estrogen and progesterone). Estrogen therapy can be used as monotherapy in the form of an oral or transdermal regime with the lowest effective dose in women with a history of hysterectomy. Combination therapy is used in women with intact uterus as the addition of progesterone protects the endometrium against the unopposed proliferating effect of estrogens. Other options that could be used include selective estrogen receptor modulators (SERMs) and tibolone. SERMs can be used to treat menopausal symptoms and prevent osteoporosis in women with contraindications or intolerance to estrogen or combination therapy. Tibolone is a synthetic steroid with estrogenic, progestin, and androgenic activity to treat menopausal symptoms only [50].

The benefits of HRT associated with improved cognition are linked to the neuroprotective effects of estrogen by directly acting on the brain or indirectly through improving patients' cardiovascular system [52]. There is evidence of an association between HRT and the risk of neurodegenerative disease, and it has been stated that estrogen formulations, specifically 17β-estradiol, are associated with reduced risk of AD and minimized cognitive decline, which particularly includes verbal memory in the otherwise healthy younger postmenopausal age group from 50 to 62 years old. No benefit of E2 was observed in the older age group [6,52].

The Women's Health Initiative Memory Study (WHIMS) is the largest randomized controlled trial evaluating the incidence of dementia in postmenopausal women taking estrogen plus progestin. The WHIMS displays that for women 65 years or older, the use of HRT has been associated with an increased risk of developing dementia and did not prevent mild cognitive impairment. The study concluded that the risks of HRT in preventing dementia override its benefits [53]. The use of HRT in midlife or the window period of menopause has proved to be beneficial in the cognitive domain. Still, the use of HRT after the age of 61 poses an increased risk of dementia. Therefore, estrogen therapy seems to be more related to maintaining rather than increasing cognitive function [54,55].

Lifestyle interventions and personalized medicine

Decreased estrogen levels in menopause are associated with reduced basal metabolism, increased body weight, and a shift in fat distribution toward more visceral fat accumulation. Consequently, menopause serves as a risk factor for obesity and metabolic syndrome. It leads to chronic inflammation systemically and in the brain [56]. The World Health Organization has recognized diet as essential in reducing the risk of cognitive decline and dementia [57]. Although there is no direct evidence linking dietary interventions to a reduced risk of AD specifically, there is evidence supporting the role of diet and supplements in preventing dementia and cognitive decline, which are critical aspects of AD [58]. Recent studies have identified distinct differences in the gut microbiome of patients with AD, suggesting a promising avenue for further research. These findings could have significant implications for lifestyle interventions and personalized medicine for individuals affected by this condition [59,60].

Adhering to a Mediterranean diet is recommended as it decreases the risk of mild cognitive impairment and AD in individuals with normal cognitive function at baseline. Adopting this diet can lower the risk of cognitive impairment and AD in postmenopausal women [61,62].

There is a lot of debate on the use of phytoestrogens for decreasing the risk of AD. Phytoestrogens are heterocyclic phenols found in plants with a structure like estrogens. Isoflavones (soy) have been widely studied. These compounds mimic some of estrogen's effects on the central nervous system. The antioxidant properties of phytoestrogens may prevent neurodegeneration, a key phenomenon in Alzheimer's. High isoflavone intake can improve processing speed in Asian women during late perimenopause and post-menopause. However, it does not have any beneficial effect on verbal memory scores in both Asian and non-Asian women [63].

Adding exercise to the daily regimen lowers the risk of Alzheimer's. Higher aerobic fitness levels are associated with increased hippocampal volumes, resulting in better memory function in the elderly [64]. Walking for 30 minutes thrice weekly for six weeks is recommended to improve complex information processing (executive function). A regimen of mixed strength and aerobic exercises (once weekly, starting at 15 minutes and gradually increasing to 45 minutes per week) for six months can decrease the risk of global cognitive decline in women. The mixed regimen should include stretching, weight training, and aerobic components such as staircase exercises [65]. Pilates has also proven beneficial for postmenopausal women due to its benefits in enhancing physical and mental functioning, promoting independence, improving sleep quality, and reducing fatigue, depression, and anxiety [66].

Mentally stimulating exercises have a beneficial effect on brain aging [67,68]. Older individuals who regularly participate in diverse cognitive activities exhibit cerebral amyloid levels comparable to those of young adults. In contrast, those who engage the least show amyloid levels like those observed in patients with AD [69]. Involvement in cognitive activities leads to more significant gray matter volumes in regions susceptible to AD pathology [64]. Evidence suggests that Kundalini yoga offers multiple benefits for cognition and memory in older women at risk of AD, including restoring neural pathways, preventing brain matter decline, and reversing biomarkers associated with aging and inflammation [65].

Future directions and research gaps

While considerable research has been conducted on the risk of AD during menopause, many questions remain unanswered. There is still a lack of evidence linking low estrogen levels and neuroinflammation to the development of Alzheimer’s. Additionally, research on brain structural changes during early menopause is limited, and evidence on the impact of estrogen use on Alzheimer’s risk is inconsistent. The relationship between the timing of HRT and Alzheimer's risk is also not well understood, with existing literature showing contradictory findings. More studies are needed to determine which types of HRT may be effective in reducing Alzheimer's risk [70].

Longitudinal studies are essential to investigate how hormonal changes during menopause correlate with cognitive decline and AD risk over time and to evaluate the long-term effects of HRT on cognitive function, considering factors such as timing, duration, and individual differences. Interventional trials should be conducted to assess the effectiveness of HRT and alternative non-hormonal interventions, such as lifestyle modifications and dietary supplements, in reducing AD risk. Biomarker research is crucial to develop and validate non-invasive biomarkers for the early detection of AD risk in menopausal women and to explore the molecular mechanisms by which estrogen provides neuroprotection.

Personalized medicine approaches are needed to create tailored AD prevention strategies based on individual hormonal, genetic, and lifestyle factors and to investigate the genetic and epigenetic influences on AD risk [71,72]. Additional research is required to explore how lifestyle interventions such as dietary changes, exercise, and cognitive training can help prevent Alzheimer’s during menopause. Multidisciplinary approaches should be fostered to encourage collaborative research across neuroscience, endocrinology, geriatrics, and psychology, aiming to develop comprehensive intervention strategies and train new medical professionals in the complexities of AD, its relationship with social factors, and their impact on quality of life [73].

## Conclusions

This review has explored the relationship between AD and the use of HRT for menopausal patients. Estrogen directly impacts brain health, improving cholinergic activity, reducing neuronal loss, and decreasing Aβ deposition. The literature is broad about the positive impact of estrogen on brain cells. Factors that contribute to the reduction in the incidence of Alzheimer's disease in post-menopausal women include engaging in personalized cognitive training programs and providing multimodal treatment approaches. Understanding the interplay between menopause and AD is crucial to emphasize the need for customized risk-benefit assessments before initiating HRT. Since there is a physiologic impact of estrogen on the brain that has not been reproduced in clinical trials, it is fundamental to continue to research this topic to provide more robust recommendations to menopausal patients about the influence of HRT on AD.
